# Lineage switch in childhood leukemia

**DOI:** 10.1002/hem3.70528

**Published:** 2026-11-04

**Authors:** Katharina Daugs, Anna Prazmo, Anindita Roy, Arndt Borkhardt

**Affiliations:** ^1^ Department of Paediatrics University of Oxford Oxford United Kingdom; ^2^ Department of Pediatric Oncology, Hematology and Clinical Immunology, Medical Faculty Heinrich Heine University Düsseldorf Düsseldorf Germany; ^3^ MRC Molecular Haematology Unit, Weatherall Institute of Molecular Medicine University of Oxford Oxford United Kingdom; ^4^ Department of Haematology Great Ormond Street Hospital for Children London United Kingdom

## Abstract

Lineage switch (LS) is a rare but aggressive immunophenotypic transformation of acute leukemia associated with limited therapeutic options and poor clinical outcome. Although LS has been increasingly recognized in the era of CD19‐directed immunotherapies, its biological basis remains incompletely understood. Most reported pediatric LS cases occur in *KMT2A*‐rearranged (*KMT2A*‐r) leukemia, highlighting a link between developmental origin and lineage plasticity. In this review, we provide a comprehensive overview of the clinical, molecular, epigenetic, and cellular determinants of pediatric LS. We systematically reviewed the literature and identified 201 pediatric LS cases reported between 1994 and 2026. Among these, 70.1% involved B‐lymphoblastic leukemias switching to acute myeloid leukemias, with *KMT2A*‐r (47.8%) and *DUX4*‐rearranged (14.9%) leukemias representing the most frequent genetic subtypes. Across the published cases, LS occurred earlier following blinatumomab exposure than after chimeric antigen receptor cells or chemotherapy alone. We discuss current mechanistic concepts underlying LS, including epigenetic remodeling, chromatin regulation, developmental programs, the cell‐of‐origin, and therapy‐associated selective pressures that promote lineage plasticity. By integrating clinical evidence with current insights into leukemia biology, this review provides a framework for understanding LS in pediatric leukemias and highlights priorities for future research, early detection, and development of preventive and/or therapeutic strategies.

## INTRODUCTION

Over recent decades, treatment of pediatric acute lymphoblastic leukemia (pALL) has achieved remarkable success, with overall survival rates >90% in high‐income countries.^1^, ^2^, ^3^ Nevertheless, rare but biologically aggressive escape mechanisms continue to limit durable cure in some cases. Among these, lineage switch (LS) represents a particularly challenging conversion of an acute leukemia from one hematopoietic lineage to another. LS may occur during treatment, at relapse, or with increasing frequency following the introduction of immunotherapy. Although uncommon, LS is associated with treatment resistance and limited standardized therapeutic strategies.^4^, ^5^, ^6^, ^7^ Consistent with its highly aggressive biology, clinical outcomes following LS remain exceedingly poor. Median overall survival following LS across all age groups has been reported to range from only 2.9 to 4.8 months.^7^, ^8^

LS is accompanied by morphological and immunophenotypic transformation while retaining the original cytogenetic or molecular lesion, thereby confirming clonal continuity and distinguishing LS from therapy‐related secondary malignancies.^7^ LS should be clearly distinguished from lineage drift (LD). LD refers to partial immunophenotypic changes in leukemic cells, characterized by the gain or loss of lineage‐associated markers, reflecting leukemic lineage plasticity but without fulfilling criteria for a complete LS, and therefore without reclassification into a different leukemic lineage.^7^, ^8^

Although LS has gained particular attention in the era of CD19‐directed immunotherapy, it is not a novel phenomenon. Cases were first described in patients relapsing after conventional chemotherapy long before the introduction of targeted immunotherapy.^9^

In unselected pediatric cohorts, LS occurs in fewer than 1% of cases but is more common in relapsed disease, where the incidence is approximately 6%–9%.^4^, ^5^, ^10^ This incidence increases to approximately 7% in leukemias harboring *KMT2A*‐ rearrangements (*KMT2A*‐r)^11^ and rises further to nearly 20% at relapse in this subgroup.^12^, ^13^ *KMT2A*, located at chromosome 11q23.3, encodes the histone‐lysine *N*‐methyltransferase 2A and is recurrently rearranged in both pALL and pediatric acute myeloid leukemia (AML).^14^ This predisposition is particularly pronounced in infants, in whom *KMT2A*‐r is detected in up to 80% of B‐lymphoblastic leukemia (B‐ALL) cases, and lineage plasticity is a defining biological characteristic.^4^, ^10^, ^15^, ^16^ *KMT2A*‐r B‐ALL frequently exhibits baseline myeloid antigen expression and was historically termed mixed‐lineage leukemia (MLL), a designation that may more accurately capture the intrinsic lineage plasticity and clinical behavior of these leukemias.^7^ In accordance with current nomenclature standards, the term *KMT2A* will be used throughout this review.

However, LS is not confined to *KMT2A‐*r leukemia and is increasingly recognized in the context of targeted immunotherapies.^17^ Beyond *KMT2A‐*r, a broader spectrum of genetic alterations has been implicated, including *DUX4*‐rearranged (*DUX4‐*r) leukemias, which appear to exhibit a predisposition toward monocytic LS, and *ZNF384*‐rearranged (*ZNF384*‐r) leukemias, which are intrinsically characterized by lineage ambiguity.^8^, ^18^, ^19^ The lineage infidelity of *DUX4*‐r combines with some uncommon clinical behavior, a notably slow MRD response reflecting relatively poor initial treatment response with classical 3‐drug or 4‐drug induction, but still excellent long‐term survival.^19^

The pathophysiology of LS remains incompletely understood, and several mechanistic models have been proposed. These include therapy‐induced selection of a pre‐existing minor subclone with an alternative lineage identity, reprogramming of a multipotent leukemic progenitor, and dedifferentiation of a lineage‐committed B‐cell precursor followed by acquisition of a new differentiation program.^4^, ^15^, ^16^, ^20^, ^21^, ^22^

Accordingly, this review provides a narrative comparison of the genetic subtypes, preceding therapeutic exposures, and immunophenotypic classifications of pediatric acute leukemia reported to undergo LS. We subsequently discuss the role of epigenetic regulators and chromatin‐remodeling pathways in LS pathogenesis, integrating insights into the cell‐of‐origin, fetal developmental programs, developmental‐stage−specific vulnerabilities, and cooperating secondary mutations with frontline treatment strategies and therapy‐associated selective pressures. Given the increasing frequency of LS in the era of CD19‐directed immunotherapy,^7^, ^17^ we specifically address cases arising after blinatumomab and chimeric antigen receptor (CAR)‐T cell therapy.

## CLINICAL CHARACTERISTICS

To characterize the clinical features of LS in pediatric leukemia, we performed a literature review using PubMed research database with the search terms “pediatric leukemia” and “lineage switch.” Of 108 records identified (last accessed May 27, 2026), 49 studies and case reports, published between 1994 and 2026, were included in the analysis.^4^, ^5^, ^6^, ^7^, ^8^, ^11^, ^12^, ^13^, ^16^, ^17^, ^23^, ^24^, ^25^, ^26^, ^27^, ^28^, ^29^, ^30^, ^31^, ^32^, ^33^, ^34^, ^35^, ^36^, ^37^, ^38^, ^39^, ^40^, ^41^, ^42^, ^43^, ^44^, ^45^, ^46^, ^47^, ^48^, ^49^, ^50^, ^51^, ^52^, ^53^, ^54^, ^55^, ^56^, ^57^, ^58^, ^59^, ^60^, ^61^ Across these publications, 201 individual pediatric LS cases were extracted. While this cohort represents, to the best of our knowledge, the most comprehensive compilation of published pediatric LS cases reported to date, the observed frequencies and subtype distributions should be interpreted with caution, as they may be influenced by publication bias, underreporting, and heterogeneous case characterization across studies. Eligible publications comprised case reports and clinical studies describing pediatric patients (0–19 years of age) with a confirmed LS from one leukemic phenotype to another. Cases of LS occurring both after initial diagnosis and at relapse were included. In patients with multiple reported LSs, only the first documented LS event was included in the analysis. Whenever available, the following variables were systematically extracted: cytogenetic and molecular alterations at initial diagnosis, additional genetic aberrations at the time of LS, immunophenotypic characterization by flow cytometry at diagnosis and LS, leukemia subtype, number of reported cases, patient sex, age at initial diagnosis, interval to LS, therapies administered before and after LS, and clinical outcome. All extracted case‐level data are provided in Table S1, and a study selection workflow is provided in Figure 1.

**Figure 1:**
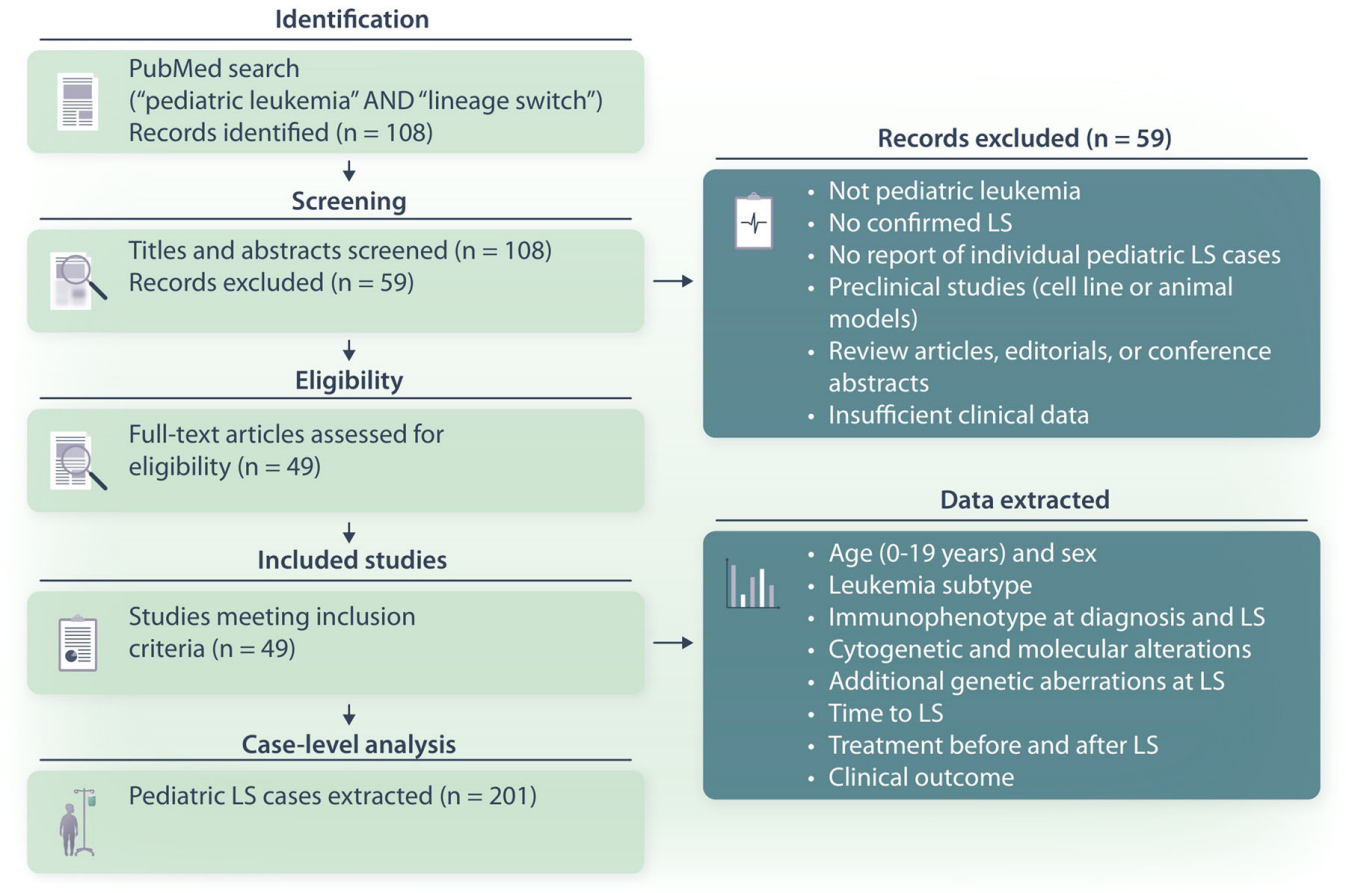
**Study selection process for the literature review of pediatric lineage switch (LS) leukemia cases.** A PubMed search using the terms “pediatric leukemia” and “lineage switch” identified 108 records (last accessed May 27, 2026). After title and abstract screening, 49 publications fulfilled the predefined inclusion criteria and were included in the qualitative synthesis. Across these studies, 201 individual pediatric LS cases were identified and included in the case‐level analysis. Extracted variables comprised demographic characteristics, leukemia subtype, immunophenotypic features, cytogenetic and molecular alterations, interval to LS, treatment history, and clinical outcome. Records were excluded if they did not report pediatric patients with a confirmed LS, represented preclinical studies (cell line or animal models), review articles, conference abstracts, or lacked sufficient clinical information for case‐level data extraction. Table S1 contains all extracted case‐level data.

The median age at initial diagnosis was 12.5 months (range, 4 days to 19 years). Based on the information available, the cohort comprises 66 infants (0–12 months), 46 children (1–19 years), 86 described as infants/children (0–18 years), and 3 adolescents and young adults (AYA) (Table 1).

**Table 1:** Patient characteristics.

Characteristics	*n* = 201
**Demographic data**	
Median age at initial diagnosis	12.5 m (4 d to 19 y)
Infants	66 (32.8%)
Children (1–19 y)	46 (22.9%)
Infants/Children (0–18 y)	86 (42.8%)
AYA	3 (1.49%)
**Sex**	
Male	40 (19.9%)
Female	31 (15.4%)
Not defined	130 (64.7%)
**Initial phenotype**	
B‐ALL (not otherwise classified)	132 (65.7%)
Pro‐B‐ALL	25 (15.1%)
Pre‐B‐ALL	5 (3.0%)
cALL	4 (2.4%)
T‐ALL/LBL	14 (7.0%)
AML	10 (5.0%)
ALAL	9 (4.5%)
MPAL	2 (1.0%)
**Flow cytometry data**	
Available	73 (36.3%)
Aberrant cross‐lineage antigen expression	29/73 (39.7%)
**Reported lineage switch**	
B‐ALL to AML	141 (70.1%)
T‐ALL/LBL to AML	12 (5.9%)
B‐ALL to MPAL/ALAL	10 (5.0%)
T‐ALL/LBL to MPAL/ALAL	3 (1.5%)
Remaining	35 (17.4%)
**Cytogenetics at initial diagnosis**	
*KMT2A*‐r	96 (47.8%)
*DUX4*‐r	30 (14.9%)
High hyperdiploid	10 (5.0%)
*ZNF384*‐r	8 (4.0%)
*PAX5* P80R	6 (3.0%)
*BCR::ABL1* positive	5 (2.5%)
*ETV6::RUNX1*	2 (1.0%)
*CRLF2*‐r	2 (1.0%)
*TLX3*‐r	2 (1.0%)
Hypodiploidy	1 (0.5%)
Philadelphia chromosome‐positive	1 (0.5%)
*MNX1::ETV6*	1 (0.5%)
Not defined subtype	37 (18.4%)
**KMT2A‐r**	*n* = 96
*KMT2A*‐r (fusions partner not defined)	49 (51.0%)
*KMT2A::AFF1*	26 (27.1%)
*KMT2A::MLLT3*	9 (9.4%)
*KMT2A::MLLT1*	6 (6.3%)
*KMT2A::AFF3*	2 (2.1%)
*KMT2A::MLLT10*	1 (1.0%)
*KMT2A::EPS15*	1 (1.0%)
*KMT2A::AFDN*	1 (1.0%)
*KMT2A::CREBBP*	1 (1.0%)

Abbreviations: ALAL, acute leukemia of ambiguous lineage; AML, acute myeloid leukemia; AYA, adolescents and young adults; B‐ALL, B‐lymphoblastic leukemia; cALL, common acute lymphoblastic leukemia; d, days; MPAL, mixed phenotype acute leukemia; T‐ALL/LBL, T‐acute lymphoblastic leukemia/lymphoma; y, years.

LS was predominantly identified within the bone marrow (BM), occurring in 198/201 cases (98.5%). However, three cases presented without BM involvement, including one isolated myeloid central nervous system leukemia,^48^ one mixed phenotype acute leukemia (MPAL) manifesting as a thoracic spinal myeloid sarcoma,^30^ and one isolated myeloid sarcoma without detectable BM disease.^54^

## LEUKEMIA PHENOTYPES

Across 201 reported cases of LS, the predominant transition was B‐ALL to AML, accounting for 141 (70.1%) cases. Additional transitions included T‐acute lymphoblastic leukemia (T‐ALL) to AML in 12 cases (5.9%), B‐ALL to MPAL/acute leukemia of ambiguous lineage (ALAL) in 10 cases (5.0%), and T‐ALL to MPAL/ALAL in 3 cases (1.5%), whereas the remaining 35 cases (17.4%) comprised less frequent phenotypic combinations.

The initial phenotype was a B‐ALL in 166/201 cases (82.6%), including pro‐B‐ALL (CD19^+^ CD10^−^ cytoplasmic immunoglobulin [cyIgµ]^−^, *n* = 25, 15.1%), pre‐B‐ALL (CD19^+^ CD10^+^ cyIgµ^+^, *n* = 5, 3.0%), common acute lymphoblastic leukemia (cALL) (CD19^+^ CD10^+^ cyIgµ^−^, *n* = 4, 2.4%), while 132 cases (79.5%) were classified as B‐ALL, not otherwise specified. The second largest subgroup comprised T‐ALL/LBL, including 13 cases of T‐ALL and one case of T‐lymphoblastic lymphoma (T‐LBL),^48^ accounting for 14 cases (7.0%), followed by AML in 10 cases (5.0%), ALAL in 9 cases (4.5%), and MPAL in 2 cases (1.0%). The phenotypic transition is shown in Figure 2.

**Figure 2:**
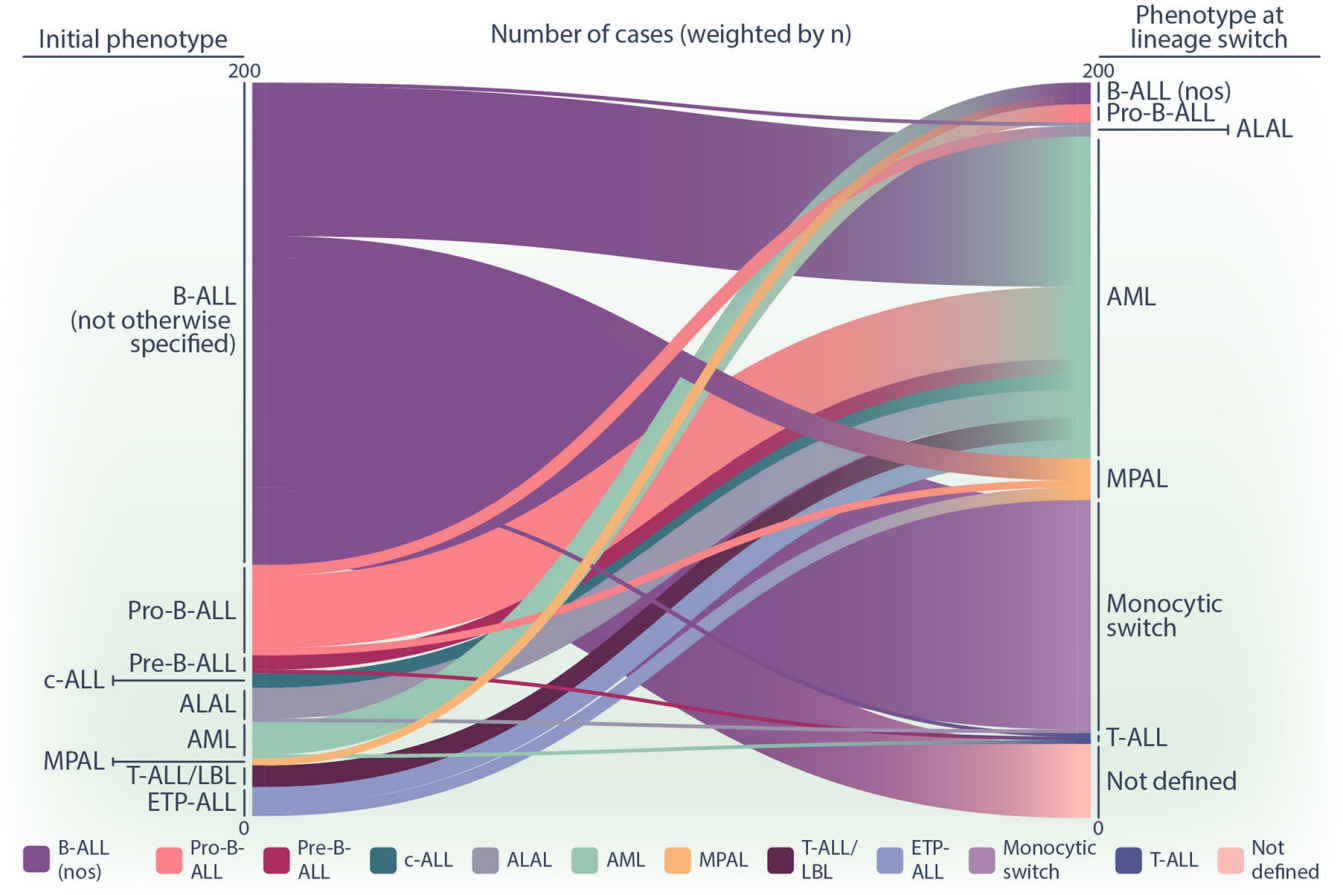
**Phenotypic transitions in lineage switch (LS) in reported pediatric acute leukemia cases.** Alluvial plot illustrating transitions between initial leukemia phenotype and phenotype observed at timepoint of LS among reported pediatric acute leukemia cases (*n* = 201; including 1 case with T‐cell lymphoma). The width of each flow is weighted according to the number of reported cases. Most LS originated from B‐lymphoblastic leukemia (B‐ALL) and predominantly transitioned toward acute myeloid leukemia (AML), or monocytic phenotypes. Subclassification of B‐ALL into pro‐B‐ALL, pre‐B‐ALL, and common acute lymphoblastic leukemia (cALL) was performed according to reported flow cytometric immunophenotypes, including CD19, CD10, and cytoplasmic immunoglobulin (cyIgµ) expression. Cases lacking detailed flow cytometric characterization were grouped as B‐ALL not otherwise specified (nos) (B‐ALL‐nos [*n* = 132], pro‐B‐ALL [CD19^+^ CD10^−^ cyIgµ^−^, *n* = 25], pre‐B‐ALL [CD19^+^ CD10^+^ cyIgµ^+^, *n* = 5], cALL [CD19^+^ CD10^+^ cyIgµ^−^, *n* = 4], T‐lymphoblastic leukemia/lymphoma [T‐ALL/LBL] [*n* = 14], AML [*n* = 10], acute leukemia of ambiguous lineage [ALAL] [*n* = 9], and mixed phenotype acute leukemia [MPAL] [*n* = 2]). AYA, adolescents and young adults; C, chemotherapy; CAR, chimeric antigen receptor; ETP‐ALL, early T‐cell precursor acute lymphoblastic leukemia.

## GENETICS

The distribution of cytogenetic and molecular subtypes at diagnosis in a large pediatric B‐ALL cohort of patients enrolled in the UKALL2003 trial is shown in Figure 3A (*BCR::ABL1* positive B‐ALL excluded).^2^, ^3^ In comparison, the cytogenetic and molecular features of patients at LS show enrichment for rare subtypes (Figure 3B). *KMT2A*‐r leukemia was the predominant subtype among reported pediatric LS cases, accounting for 96 of 201 cases (47.8%), with *KMT2A::AFF1* representing the most frequent fusion partner (*n* = 26, 27.1% of *KMT2A*‐r cases). Additional recurrent alterations included *DUX4*‐r (*n* = 30, 15.0%), high hyperdiploid (*n* = 10, 5.0%), *ZNF384*‐r (*n* = 8, 4.0%), and *PAX5* P80R (*n* = 6, 3.0%). For 37 cases (18.4%), no informative genetic data were available at initial diagnosis, reflecting either the absence of genetic testing, incomplete reporting, or the lack of detectable recurrent genetic alterations. Moreover, comprehensive flow cytometric and/or immunohistochemical characterization at both initial diagnosis and at timepoint of LS was available for 73/201 cases (36.3%). Aberrant cross‐lineage antigen expression at initial diagnosis was observed in 29 of 73 evaluable cases with available flow cytometry (39.7%), corresponding to 29 of 201 overall cases (14.4%) (see Tables 1 and S1).

**Figure 3:**
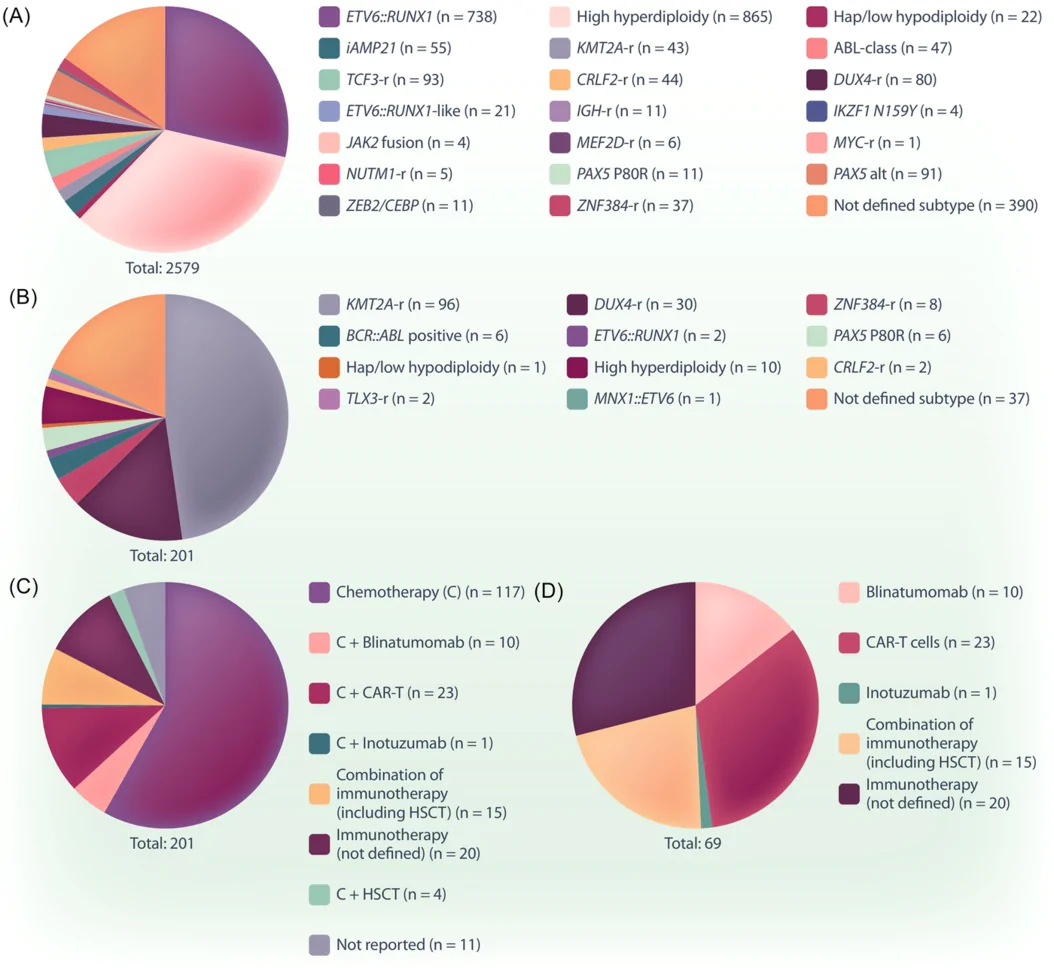
**Cytogenetic subtype distribution and therapy exposure before lineage switch (LS) in reported pediatric acute leukemia cases. (A)** Distribution of cytogenetic and molecular subtypes among pediatric B‐lymphoblastic leukemia (B‐ALL) cases enrolled in the UKALL2003 clinical trial (genetics for *n* = 2579 B‐ALL patients extracted), adapted from Vora et al.^2^ and Kirkwood et al.^3^ (*BCR::ABL1* positive ALL excluded). Abbreviation: r, rearrangement. **(B)** Pie chart showing the distribution of cytogenetic subtypes at initial diagnosis among reported pediatric acute leukemia cases with LS (*n* = 201). Compared with the general pediatric B‐ALL population, **(A)** *KMT2A*‐r leukemia is substantially overrepresented among reported pediatric LS cases. **(B)** Cases without sufficient molecular information were classified as “not defined subtype.” **(C)** Distribution of therapies administered prior to development of LS (*n* = 201). Cases lacking detailed treatment information were classified as “not reported.” Abbreviation: C, chemotherapy. **(D)** Summary of proximal immunotherapy exposure before development of LS among patients receiving immunotherapy‐associated treatment regimens (*n* = 69). Cases in which the exact immunotherapeutic agent was not specified in the original report were grouped as “immunotherapy not defined.” Combination regimens included combined immunotherapeutic approaches and/or hematopoietic stem cell transplantation (HSCT). CAR, chimeric antigen receptor.

## SECONDARY GENETIC MUTATIONS

Additional mutations, for example, KRAS, NRAS, or PTEN mutations were seen in 18 cases (9.0%) at initial diagnosis, increasing to 32 (15.9%) at LS. However, these frequencies are likely underestimated, as comprehensive molecular analyses were not available or reported for all cases, particularly in older studies. Consequently, the absence of reported secondary mutations should not be interpreted as their true absence. Emerging evidence suggests that these lesions do not directly induce lineage conversion but rather promote genomic instability, clonal fitness, and adaptive stress tolerance, thereby facilitating therapy‐driven selection and expansion of pre‐existing plastic subclones. Reported alterations most frequently involved the RAS pathway (*KRAS*, *NRAS*, *PTPN11*, and *FLT3*) and the PI3K pathway (*PTEN*), although the true prevalence of these lesions remains uncertain because of heterogeneous molecular testing and reporting across the included studies. For example, *TP53* alterations have been associated with antigen‐negative relapse following CD19‐directed therapy, whereas activating RAS‐pathway mutations may reinforce stemness‐associated and myeloid transcriptional programs during lineage transition.^8^, ^62^, ^63^, ^64^

## THERAPY AND OUTCOME

The 201 pediatric LS cases included in this analysis were reported between 1994 and 2026, spanning substantial changes in leukemia diagnostics, risk stratification, and therapeutic approaches. Consequently, the reported treatment exposures preceding LS should be interpreted within their historical context, as the use of contemporary immunotherapies, including bispecific T‐cell engagers (BiTEs), antibody–drug conjugates, and CAR‐T cell therapies, was limited or unavailable during much of this period and may not reflect current frontline treatment practices. Therapeutic exposures preceding LS in the 201 individual cases of LS were heterogeneous. Chemotherapy alone was administered in 117 patients (58.2%). Blinatumomab alone was added to chemotherapy in 10 cases (5.0%), CAR‐T cells were added to chemotherapy in 23 cases (11.4%), and inotuzumab ozogamicin alone was added to chemotherapy in one patient (0.5%). Four patients (2.0%) received combined immunotherapeutic approaches prior to LS. Twenty patients (10.0%) were reported to have received immunotherapy before LS without further specification of the exact agent, including blinatumomab, CAR‐T cells and inotuzumab ozogamicin. Four patients (2.0%) underwent hematopoietic stem cell transplantation (HSCT) without immunotherapy prior to LS. 11 patients (5.5%) underwent HSCT in combination with immunotherapy prior to LS. Overall, 69 patients (34.3%) received at least one form of immunotherapy prior to LS. Detailed information regarding treatment exposure before LS was unavailable in 11 cases (5.5%). Therapy exposure before LS can be found in Figure 3C,D and Table 2 and S1.

**Table 2:** Therapy and outcome.

Characteristics	*n* = 201
**Therapy prior lineage switch**	
Chemotherapy (C) alone	117 (58.2%)
C + blinatumomab	10 (5.0%)
C + CAR‐T cells	23 (11.4%)
C + inotuzumab ozogamicin	1 (0.5%)
Combined immunotherapy	4 (2.0%)
Immunotherapy non‐defined	20 (10%)
HSCT without immunotherapy	4 (2.0%)
HSCT with immunotherapy	11 (5.5%)
Not reported	11 (5.5%)
**Therapy after lineage switch**	
Chemotherapy (C) alone	33 (16.4%)
C + HSCT	23 (11.4%)
C + HSCT + immunotherapy	5 (2.5%)
C + immunotherapy	6 (3.0%)
Palliative care alone	12 (6.0%)
Not reported	122 (60.7%)

**Reported interval between last therapeutic intervention and lineage switch**	Median (min–max, months)	mean ± SD, months
Overall	3.0 (0.4–48) (*n* = 73)	8.54 ± 11.26
Following blinatumomab	0.9 (0.5–5.0) (*n* = 11)	1.47 ± 1.53
Following CAR‐T cells	2.2 (1–36.0) (*n* = 24)	5.93 ± 7.97
Following chemotherapy	8.4 (0.4–48.0) (*n* = 38)	12.22 ± 13.17
**Survival after lineage switch**		
Dead	100 (49.8%)	
Alive	24 (11.9%)	
Not reported	77 (38.3%)	

*Note*: The treatment‐to‐Lineage switch (LS) interval was based on timing reported in the original publications. For chemotherapy‐treated patients, the exact timing of individual chemotherapy cycles relative to LS was not consistently available.

Abbreviations: CAR, chimeric antigen receptor; HSCT, hematopoietic stem cell transplantation.

Among patients treated with chemotherapy, blinatumomab, or CAR‐T cell therapy and included in the treatment‐specific time‐to‐LS analysis, the median reported interval between last therapeutic intervention and LS was 3.0 months (range, 0.4–48 months). Time to LS differed significantly between treatment groups. Patients with LS following blinatumomab therapy showed the shortest interval to LS, with a median time of 0.9 months (range, 0.5–5 months); (Table 2). Post hoc Dunn's multiple comparisons testing demonstrated significantly shorter time to LS after blinatumomab than after CAR‐T cell therapy or chemotherapy (Figure 4A).

**Figure 4:**
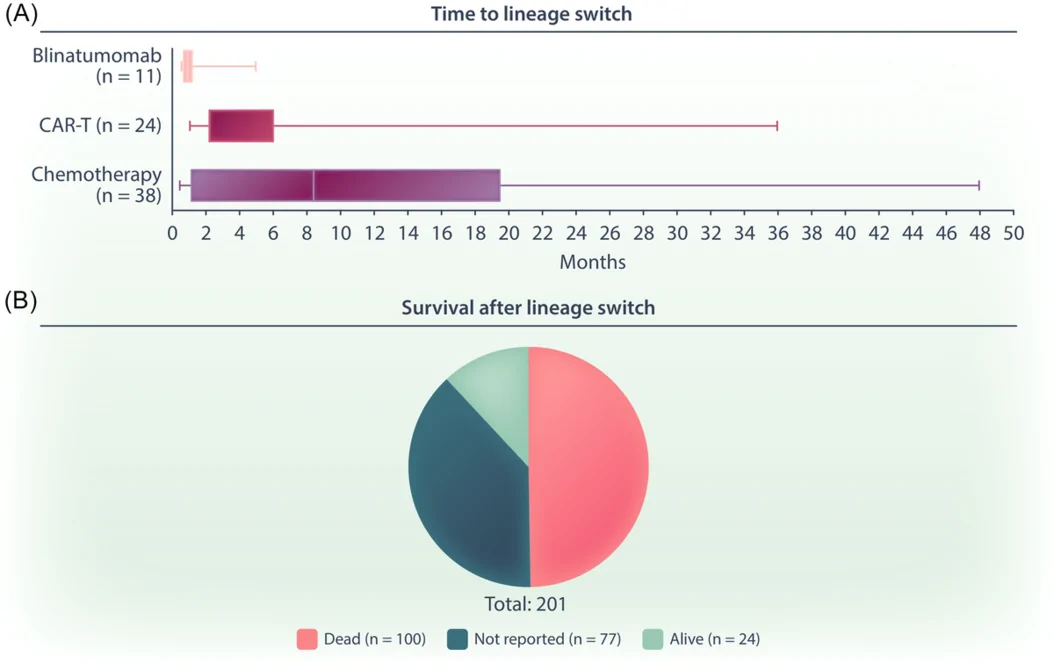
**Interval between last therapeutic intervention and Lineage switch (LS) and survival outcomes following LS in reported pediatric acute leukemia cases. (A)** Box‐and‐whisker plots showing the reported interval between therapeutic intervention and LS among patients treated with chemotherapy (*n* = 38), chimeric antigen receptor (CAR)‐T cell therapy (*n* = 24), or blinatumomab (*n* = 11). Boxes represent the median and interquartile range (IQR), and whiskers represent minimum and maximum values. Time to LS differed significantly between treatment groups (Kruskal–Wallis, P < 0.0001). Post hoc Dunn's multiple comparisons testing demonstrated a significantly shorter interval following blinatumomab compared with CAR‐T cell therapy (adjusted P = 0.0222) and chemotherapy (adjusted P = 0.0026). These findings should be interpreted with particular caution given the small number of patients in each treatment group, the restriction to published cases, and the heterogeneous reporting of treatment timing, particularly for chemotherapy. **(B)** Pie chart illustrating survival status after LS among reported pediatric acute leukemia cases (*n* = 201). Approximately half of the reported patients deceased following LS (*n* = 100), while only a minority were reported alive (*n* = 24) at last follow‐up. Outcome data were unavailable or not reported for 77 cases.

Following LS diagnosis, therapeutic management was highly heterogeneous and largely guided by individual clinical judgment, reflecting the lack of established treatment standards. Approaches ranged from chemotherapy alone to multimodal regimens incorporating HSCT and immunotherapy. Twelve patients (6.0%) received palliative care without further disease‐directed treatment. Detailed treatment and outcome data, when available, are provided in Table S1.

Overall, 24 patients (11.9%) achieved disease remission following LS and remained alive at the last reported follow‐up. In contrast, 100 patients (49.8%) deceased, predominantly due to progressive or refractory disease. Outcome data, however, were incompletely reported for 77 patients (38.3%), limiting comprehensive survival analyses (Figure 4B).

Among the 24 patients reported alive after LS, detailed post‐LS treatment information was unavailable in 4 cases. Of the remaining 20 patients, 13 (65.0%) achieved long‐term remission following HSCT. Targeted therapies were incorporated into the treatment regimen in 3 patients, including revumenib (*n* = 1), flavopiridol (*n* = 1), and sorafenib (*n* = 1). In addition, gemtuzumab ozogamicin (*n* = 2) and mitoxantrone (*n* = 2) were components of successful therapeutic approaches. Overall, most treatment strategies were based on intensive high‐dose cytarabine–containing regimens, reflecting the predominance of myeloid phenotypes after LS. Detailed information regarding therapy exposure after LS can be found in Table S1.

## CELL‐OF‐ORIGIN AND DEVELOPMENTAL CONTEXT

Whether a leukemia can switch lineage can be linked to the properties of the cell in which it arose. We mainly discuss this in the context of LS in B‐ALL, and these are summarized in Figure 5. In adult BM, B‐lineage commitment proceeds from self‐renewing hematopoietic stem cells (HSCs; Lineage^−^CD34^+^CD38^−^CD45RA^−^CD90^+^) through multipotent progenitors (MPPs; Lineage^−^CD34^+^CD38^−^CD45RA^−^CD90^−^), lymphoid‐primed multipotent progenitors (LMPPs; Lineage^−^CD34^+^CD38^−^CD127^−^CD45RA^+^), and common lymphoid progenitors (CLPs; CD34^+^CD10^+^CD19^−^) to the first CD19^+^, B‐committed progenitors (CD34^+^CD10^+^CD19^+^) and finally the mature B‐cell (CD34^−^CD19^+^).^65^ Detailed analysis of human fetal B‐lymphopoiesis additionally identified two states that are largely restricted to fetal life: an early lymphoid progenitor (ELP; CD34^+^ CD38^+^CD127/IL‐7R^+^CD10^−^CD19^−^) with combined B‐, natural killer (NK)‐, T‐lymphoid and some residual myeloid potential, and a CD10^−^ pre‐pro‐B‐progenitor (CD34^+^CD10^−^CD19^+^) that represents the earliest B‐lineage‐restricted cell in human fetal life. These fetal‐specific progenitors have an ontogeny‐specific transcriptional program not seen in its rare adult counterpart.^65^, ^66^ Human fetal ELP and pre‐pro‐B‐progenitors are transcriptionally similar to CD10^−^‐*KMT2A*‐r infant ALL (iALL) blasts, suggesting these developmental‐stage–specific features may play a key role in the biology of leukemia.^66^ Two variables may determine LS propensity, the developmental position of the cell‐of‐origin in the hematopoietic hierarchy, and the developmental/ontogeny window (fetal vs. postnatal) in which transformation occurs, and below we discuss both.

**Figure 5:**
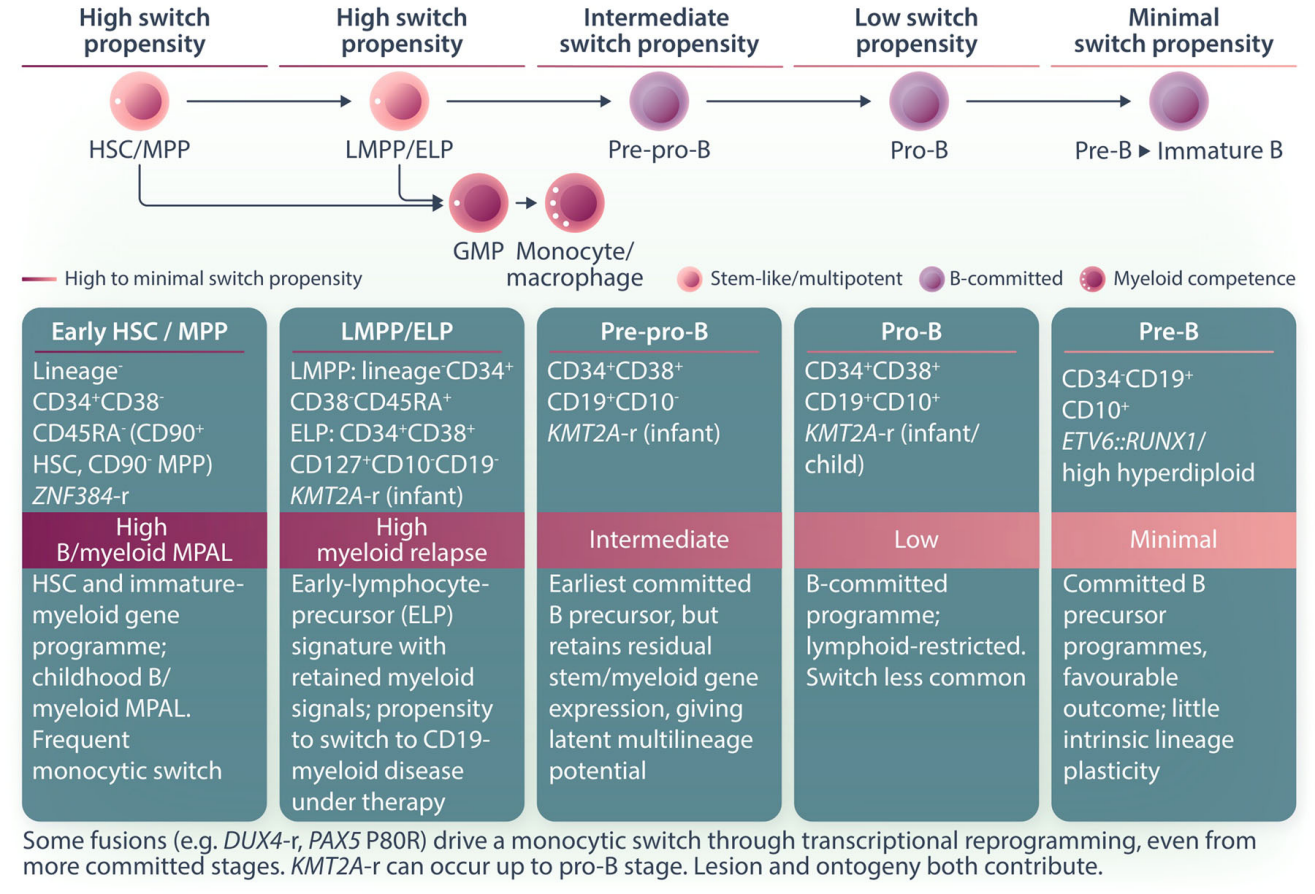
**Cell‐of‐origin and lineage‐switch (LS) propensity in B‐lymphoblastic leukemia (B‐ALL).** Candidate cells of origin are arranged along the normal fetal B‐lymphoid hierarchy (hematopoietic stem cell [HSC]/multipotent progenitor [MPP] → lymphoid‐primed multipotent progenitor [LMPP]/early lymphoid progenitor [ELP] → pre‐pro‐B → pro‐B → pre‐B/immature B). The color grades the proposed propensity to switch from high (left) to minimal (right); branches to the granulocyte–macrophage progenitor (GMP) and monocyte/macrophage show the myeloid output retained by the earlier stages. Colors denote a stem‐like/multipotent state, B‐commitment, and myeloid competence. Cell types are described as follows: HSC/MPP, Lineage^−^CD34^+^CD38^−^CD45RA^−^ (CD90^+^ HSC, CD90^−^ MPP); LMPP, Lineage^−^CD34^+^CD38^−^CD45RA^+^ and ELP, CD34^+^CD38^+^CD127^+^CD10^−^CD19^−^, retaining myeloid potential; pre‐pro‐B, CD34^+^CD38^+^CD19^+^CD10^−^; pro‐B, CD34^+^CD38^+^CD19^+^CD10^+^; pre‐B, CD34^−^CD19^+^CD10^+^, maturing to immature B, CD34^−^CD19^+^. These compartments are increasingly recognized as a continuum rather than discrete gates, and there is some heterogeneity within each population. The lower panels link each state to its associated subtypes. Propensity is highest at the earliest stages: an HSC/MPP‐like state and an LMPP/ELP‐like state (*ZNF384*‐r and infant *KMT2A*‐r) retain myeloid gene expression. The pre‐pro‐B state retains residual stem and myeloid expression (intermediate propensity); the pro‐B (low) and pre‐B‐like stages (*ETV6::RUNX1*, high hyperdiploid; minimal) follow committed B‐precursor programs with little plasticity. Some lesions (*DUX4‐*r, *PAX5* P80R) drive a monocytic switch from more committed stages, and *KMT2A‐*r can arise up to the pro‐B stage. Lesion and ontogeny both contribute. MPAL, mixed phenotype acute leukemia; r, rearrangement.

iALL originates *in utero* and is prone to LS, suggesting that features of fetal hematopoietic stem and progenitor cells (HSPCs) might provide the permissive context for LS. A switch from multipotent to largely oligo/unipotent stem cells has been documented between fetal and adult life, suggesting less lineage restriction in fetal cells.^67^ Fetal B‐progenitors are highly proliferative and express fetal‐specific genes, such as *LIN28B* and its downstream effectors, that are silenced in postnatal tissues.^65^, ^68^, ^69^ These fetal molecular programs make HSPC more permissive to transformation and are the likely reason why a single‐hit *KMT2A*‐r^70^, ^71^ in utero can give rise to early‐onset iALL, which is more aggressive than cytogenetically identical *KMT2A*‐r acute lymphoblastic leukemia (ALL) of older children.^68^, ^70^, ^72^ *KMT2A*‐r iALL demonstrates a transcriptional signature compatible with a fetal‐HSPC origin.^73^ Human fetal‐liver HSPC‐derived models of *KMT2A::AFF1* iALL are distinct from *KMT2A*‐r pALL and recapitulate the features of iALL more faithfully than equivalent postnatal HSPC‐derived models.^74^, ^75^

The progenitor cell in which leukemia arises could be important for two reasons. First, it may determine whether the leukemia retains multipotency or plasticity. Second, if the cell‐of‐origin is a CD19^−^ upstream MPP, it may escape CD19‐directed immunotherapy and drive relapse of a different lineage. HSCs or MPPs are the most primitive progenitors and retain myeloid, lymphoid and megakaryocyte–erythroid competence. LMPPs are more lymphoid‐primed, having lost megakaryocyte–erythroid potential, but remain multipotent, whereas downstream progenitors become increasingly lineage restricted. Introduction of *KMT2A::AFF1* in embryonic‐stage murine HSPCs, but not in more lineage‐committed lymphoid progenitors, gives rise to a distinctive CD24^+^ pre‐pro‐B pre‐leukemic state with multilineage potential and aberrant engraftment.^76^ In murine *KMT2A::AFF1* models, miR‐130b drives a B‐ALL/myeloid MPAL propagated by LMPP‐like cells, whereas miR‐128a drives a more lineage‐restricted pro‐B ALL.^77^

ELPs lie downstream of LMPP and are found mainly in fetal life. ELPs may be important for LS because although predominantly lymphoid, they retain some myeloid potential. In *KMT2A*‐r iALL, single‐cell transcriptomics demonstrates fetal ELP‐like profile co‐expressing lymphoid and myeloid programs, whereas the more favorable *NUTM1*‐rearranged iALL maps to later, committed B‐cell stages.^73^

The fetal pre‐pro‐B progenitor is B‐lineage restricted but has fetal‐specific gene expression programs including stem cell and non‐B lineage genes.^65^, ^66^ *KMT2A*‐r blasts resemble this cell state, and these non‐B‐lineage/stem cell programs may be upregulated during LS.

Heterogeneity of leukemic cells preserves plasticity in established disease. About 70% of B‐ALL cases contain CD34^+^CD19^−^CD22^+^ B‐cell progenitors. These cells carry the same diagnostic genetic lesion, increase in number post CD19‐directed therapy, and can propagate leukemia in NSG mice. They therefore offer a route by which the disease can escape CD19‐directed therapy.^78^ Single‐cell multi‐omics profiling of *KMT2A*‐r iALL has identified therapy‐resistant, stem‐cell‐like blasts and increased lineage plasticity, predominantly in patients younger than 6 months,^16^ and *PROM1*/CD133^+^ *KMT2A*‐r ALL blasts are a proliferative, stem‐cell‐like fraction that is more aggressive on xenotransplantation.^79^ Such subpopulations may give rise to a switched, antigen‐negative leukemia.

Mapping 89 B‐ALL samples onto a normal human B‐cell developmental atlas demonstrated the subtypes most prone to diverge from the B‐lineage: *KMT2A*‐r, *DUX4*‐r, *ZNF384*‐r, and *BCR::ABL1*‐positive disease. They exhibit MPP‐like states characterized by retained myeloid competence and *CEBPA* activation. A composite multipotency score derived from this analysis was independently associated with worse survival.^80^ The genetic lesion and the cell‐of‐origin are thus best viewed as interacting determinants, the lesion is necessary, but its lineage outcome is influenced by the developmental state of the cell it transforms and by the regulatory programs involved.

Single‐cell analysis reveals a heterogeneous maturation profile with an immature (*NFATC4*‐high) population and a more differentiated *CEBPA/FLT3*‐high population. *DUX4*‐r blasts frequently aberrantly express CD371, CD14, CD33, and CD2 with loss of B‐cell markers, reflecting a monocytic bias.^19^, ^28^ Mechanistically, the truncated DUX4 fusion protein drives aberrant transcription and splicing that affect B‐cell differentiation and promote myeloid‐associated states.^19^, ^28^, ^81^ The pre‐immunotherapy recognition of CD2^+^ and CD371^+^ B‐ALL subtypes with early monocytic switch^81^, ^82^ suggests that a LS‐permissive cell state can be present at diagnosis, before any therapeutic pressure.

Taken together, the evidence suggests that leukemias arising from more primitive cells (HSC/MPP, LMPP, and ELP) are more likely to undergo LS than those at B‐lineage‐committed stages, because such cells retain latent myeloid potential. When these progenitors are of fetal origin, the ontogenic stage of the progenitors is likely to be permissive to plasticity. It is important to emphasize that the dominant cell‐state of a leukemia at diagnosis is not the same as its cell‐of‐origin. The ELP/pre‐pro‐B‐like signature of *KMT2A*‐r iALL, for example, may reflect a downstream B‐lineage progenitor adapting an earlier developmental program or an upstream cell‐of‐origin with a transcriptional program that has evolved till the ELP/pre‐pro‐B state.^66^, ^73^ Distinguishing the properties of leukemia‐initiating cells from the leukemic cell‐state and defining how each contributes to plasticity remains to be investigated in the human fetal models developed for this purpose.^68^, ^74^, ^75^, ^76^

## TRANSCRIPTIONAL AND EPIGENETIC MECHANISMS OF LINEAGE REPROGRAMMING

The molecular mechanism of LS involves downregulation of the B‐cell transcription and activation of myeloid transcription. These processes are facilitated by epigenetic remodeling, including changes in chromatin accessibility, histone modification, and enhancer activation, that change the regulatory network and transcription factor (TF) activity of a leukemic cell.

In normal hematopoiesis, B‐cell identity is established and maintained by a hierarchy of TFs, among which PAX5 and EBF1 are particularly important. These factors maintain B‐cell identity by activating B‐lymphoid gene expression program while simultaneously repressing lineage‐inappropriate genes, including those associated with C/EBPα‐dependent myelopoiesis and T‐cell differentiation.^15^, ^83^, ^84^ Under CD19‐CAR‐T pressure, experimental deletion of *PAX5* or *EBF1* has been shown to recapitulate lineage reprogramming in murine models, confirming the need of these factors for the maintenance of lymphoid commitment when subjected to immunotherapy‐mediated selection.^83^, ^84^

Data from patient samples also implicate *PAX5* disruption in LS, with *PAX5* alterations documented as early genetic events in *KMT2A*‐r iALL that destabilize B‐cell identity and in some contexts have been shown to prime the clone for subsequent lineage conversion.^27^ In *KMT2A* wild‐type ALL LS following immunotherapy, phylogenetic reconstruction by whole‐genome sequencing has revealed a different mutational sequence in which early hemizygous loss of *PAX5* is followed by a second hit producing biallelic inactivation that precedes the divergence of the leukemic clone into lymphoid and myeloid lineages.^36^ Conversely, in some cases *PAX5* alterations are acquired at relapse, indicating that the timing of TF loss relative to therapeutic pressure is variable across patients.^27^, ^36^

An alternative mechanism of LS is based on activation of the myeloid transcriptional network, in which PU.1 (encoded by *SPI1*), C/EBPα, C/EBPβ, and RUNX1 are critical.^8^, ^15^ C/EBPα can physically displace PU.1 from B‐cell‐specific enhancers, leading to chromatin closure at lymphoid regulatory loci and concurrent activation of macrophage‐specific enhancers.^85^ C/EBPβ can reprogram fully committed B‐cells into granulocyte–macrophage progenitor (GMP)‐like populations that retain self‐renewal capacity, demonstrating that myeloid TFs are sufficient to override established lymphoid identity and that the resulting reprogrammed cells can sustain clonal expansion.^85^ The dynamics of this process is also subject to epigenetic regulation, as CARM1‐mediated arginine methylation of C/EBPα modulates the rate of lineage conversion.^85^

*KMT2A‐r* ALL LS cases demonstrate large‐scale changes in chromatin accessibility, coordinated transcriptional and alternative splicing reprogramming, and dysregulation of chromatin remodeling.^15^ Particularly important are the chromodomain helicase DNA‐binding protein 4 (CHD4) and the nucleosome remodeling and deacetylase (NuRD). Perturbation of *CHD4* alone is sufficient to induce myeloid gene expression in leukemic cells, identifying this regulator as directly involved in the maintenance of lineage identity.^15^ Specific gene rearrangements promote lineage ambiguity through related epigenetic mechanisms. *ZNF384*‐r recurrently identified in B‐ALL with mixed‐lineage features, act through enhancer remodeling and TF network dysregulation. Alterations in the *PTEN*–PU.1 axis further demonstrate that chromatin reconfiguration alone, independent of new genetic mutations, can shift lineage potential.^8^

## INFLAMMATORY AND CYTOKINE‐MEDIATED MICROENVIRONMENT

Inflammatory and cytokine‐mediated signals have been proposed as potential contributors to LS. Interleukin‐6 (IL‐6) induced irreversible myeloid differentiation of a *KMT2A::AFF1* leukemia cell line, with induction of CD33, CD13, and CD11b.^86^ Although this observation in a cell line cannot establish a microenvironmental contribution to LS in vivo, there are other lines of supporting evidence. (i) Lineage fate determination in MPPs: IL‐6 suppresses B‐lymphoid differentiation while favoring myeloid output in early uncommitted HSCs. This effect is reversible. B‐lymphoid progenitor and CD19^+^ B‐cell output is restored without IL‐6, which acts directly on multipotent progenitors via STAT3 and *ID1*.^87^ The same principle extends to malignant progenitors. In a murine chronic myelogenous leukemia model, IL‐6 redirects leukemic MPPs from lymphoid toward myeloid lineage, with the fusion oncoprotein driving IL‐6 expression.^88^ In a human *KMT2A::MLLT3* model, CD34^+^ cells cultured with stem cell factor, IL‐3, IL‐6, Fms‐like tyrosine kinase 3 ligand, and thrombopoietin, or exposed to an in vivo microenvironment providing SCF, GM‐CSF, and IL‐3, influenced leukemic lineage outcome.^89^ Notably, some of these cytokines (GM‐CSF, IL‐3, and IL‐6) overlap with the inflammatory milieu after CAR‐T cell therapy.^90^ (ii) B lineage‐to‐myeloid conversion: Inflammatory signaling also induces TFs promoting LS. Infection‐related cytokines selectively upregulate C/EBPβ in progenitors.^91^ C/EBPα and C/EBPβ are sufficient to convert B‐lineage cells to macrophages,^92^ and myeloid enhancers in lymphoid progenitor cells may persist in a state activatable by microenvironmental signals.^93^ Inflammatory signaling through TNF and Nuclear factor κ‐light‐chain‐enhancer (NF‐κB) can further induce PU.1 and promote myeloid bias and epigenetic reprogramming.^8^, ^15^

Collectively, these preclinical data provide biological plausibility that inflammatory/cytokine‐mediated signals can influence lymphoid–myeloid lineage decisions in multipotent progenitors. However, direct clinical evidence linking this to LS in leukemia remains sparse. Gardner et al.^12^ reported severe CRS with elevated IL‐6 in a *KMT2A*‐r B‐ALL patient who subsequently developed myeloid relapse. Wölfl et al.^50^ described a rapid *KMT2A*‐r LS during blinatumomab therapy accompanied by an inflammatory response, followed by spontaneous reversion after treatment discontinuation. However, the high prevalence of CRS after CAR‐T cell therapy and its close relationship with CAR‐T cell expansion argue against CRS as a major independent cause of LS, which occurs in only 2.9% of patients post CAR‐T cell therapy.^17^

Thus, inflammatory and cytokine‐mediated signaling may select for or modulate lineage plasticity in susceptible multipotent or lineage‐restricted leukemic clones rather than independently initiate LS. Whether early IL‐6 or IL‐1 inhibition alters LS incidence in *KMT2A‐r* disease, and whether myeloid‐cytokine conditions promote LS in human *KMT2A‐r* models, remain to be determined.

## DISCUSSION

This review synthesizes what is, to our knowledge, the largest body of reported pediatric LS cases available to date and integrates clinical, genomic, single‐cell, and functional data to understand LS in pediatric leukemia.

Based on the literature reviewed here, LS predominately occurs from B‐ALL to AML (70.1%), followed by T‐ALL to AML (5.9%), B‐ALL to MPAL/ALAL (5.0%), and T‐ALL to MPAL/ALAL (1.5%). These findings support those reported by Project EVOLVE, currently the largest international multicenter cohort of therapy‐associated LS, which retrospectively analyzed 75 cases across all age groups (median age 11.9 years) following antigen‐directed immunotherapy, mainly CD19‐ or CD22‐targeted treatment for B‐ALL (CAR‐T cells [48.6%], blinatumomab [44.3%], and inotuzumab ozogamicin [5.7%]). Similar to this review, the predominant transition was to AML, highlighting myeloid reprogramming.^7^

In contrast to B‐ALL‐to‐AML transformation, T‐ALL LS appears to be driven primarily by the intrinsic developmental plasticity of early thymic progenitors. Early T‐cell precursor leukemias retain stem cell‐associated and myeloid transcriptional programs, while disruption of key T‐lineage commitment regulators, including BCL11B and NOTCH1, permits reactivation of latent myeloid differentiation states.^8^, ^94^, ^95^

Furthermore, our analysis confirmed *KMT2A*‐r as a major predisposing factor for LS, present in 47.3% of reported cases (Figure 3B). Notably, *KMT2A‐r* leukemia accounted for only 43 of 2579 cases (1.67%) in the UKALL2003 pediatric B‐ALL cohort (Figure 3A),^2^, ^3^ demonstrating marked overrepresentation among reported pediatric LS cases. Additional cytogenetic and molecular alterations also drive LS; however, the mechanistic basis of LS in *ZNF384*‐r, *DUX4*‐r, *BCR::ABL1*‐positive, and CD371‐positive disease remains less well understood despite growing clinical relevance.^15^, ^80^, ^81^ Notably, *PAX5* P80R was identified in 6 of 201 (3.0%) reported LS cases, representing an apparent enrichment compared with unselected pediatric B‐ALL cohorts, including 11 of 2579 cases (0.4%) in UKALL2003^2^, ^96^ and 20 of 1028 BCP‐ALL cases (1.9%) in the AIEOP‐BFM ALL 2000 cohort.^97^ This enrichment is biologically plausible given the central role of *PAX5* in maintaining B‐cell identity and the impaired B‐cell differentiation associated with *PAX5* P80R.^98^ However, these data should be interpreted cautiously, as *PAX5* P80R was not routinely assessed in older LS reports and molecular testing varied substantially across studies. Thus, its true prevalence and association with LS remain to be established in prospectively characterized cohorts.

Although recent studies did not identify aberrant cross‐lineage antigen expression at initial diagnosis as a definitive predictor of LS,^7^, ^15^, ^73^ it was observed in 39.7% of cases in this review and may indicate lineage plasticity, warranting further investigation.

The incidence of LS following blinatumomab remains poorly defined.^23^ The incidence after CAR‐T cell therapy varies considerably across studies reaching 22% to 29% in some cohorts,^12^, ^17^, ^47^ while other cohorts have demonstrated durable remissions with only rare lineage switching.^99^, ^100^ CD19 loss appears more frequently after CD19‐directed CAR‐T cell therapy than after blinatumomab.^6^, ^23^, ^101^

Among patients included in the treatment‐specific analysis, the median reported interval between therapeutic intervention and LS was 3.0 months, with time to LS following blinatumomab and CAR‐T cell therapy similar to that reported in Project EVOLVE.^7^ Overall, patients exposed to immunotherapy appeared to develop LS earlier than those treated with chemotherapy alone (median: 8.4 months).

The rapid onset of LS following CD19‐ or CD22‐directed immunotherapy strongly supports an immune escape mechanism. Rather than inducing de novo lineage reprogramming, antigen‐directed therapies appear to selectively eliminate lymphoid leukemic populations while promoting the expansion of rare pre‐existing subclones with latent myeloid differentiation potential.^7^, ^8^, ^34^, ^102^, ^103^ In genetically plastic leukemias such as *KMT2A*‐r, this selective pressure may reveal dormant myeloid transcriptional programs, whereas chemotherapy‐associated LS likely reflects a slower process of clonal adaptation and progressive lineage remodeling.^7^, ^8^, ^34^, ^102^, ^103^ This model is supported by reports of LS occurring within days of blinatumomab initiation and by the reversible nature of LS observed in some *KMT2A*‐r iALL cases following treatment withdrawal.^6^, ^50^, ^104^, ^105^

Together, these findings suggest that immunotherapeutic pressure alone is insufficient to drive LS and instead acts on a permissive biological environment defined by intrinsic leukemic plasticity, developmental state, and clonal heterogeneity.^100^, ^106^ Notably, concurrent CD19/CD22 targeting may constrain LS escape by eliminating transitional CD22^+^ leukemic states.^83^

In total, 13 of 20 patients (65.0%) with LS in this review achieved long‐term remission following HSCT, suggesting that consolidative transplantation may support successful LS management. These findings are consistent with Project EVOLVE^7^ and other reports recommending consolidative HSCT for post‐immunotherapy relapse.^23^, ^107^ Targeted therapies for LS have shown variable results. Case reports suggest variable responses to menin‐inhibitors in *KMT2A*‐r leukemia,^11^, ^39^ while 6 patients treated with menin‐inhibitors in Project EVOLVE failed to achieve complete remission.^7^ Importantly, LS arising during menin‐inhibitor treatment has also been reported.^108^ In contrast, Oszer et al. reported successful LS treatment using CAR‐T cell therapy combined with sorafenib targeting a persistent *FLT3* mutation, followed by sorafenib maintenance therapy.^46^ Although conclusions are limited by small numbers, these observations suggest that targeted therapies may help to overcome LS, particularly integrated into post‐transplant maintenance.

We propose that a developmentally immature leukemic clone, probably arising in fetal or early postnatal hematopoiesis and frequently characterized by *KMT2A‐*r, retains a transcriptional state with multilineage potential.^16^, ^70^, ^73^, ^74^, ^75^, ^76^, ^79^, ^80^ This state alone may facilitate LS. Antigen‐directed immunotherapy then acts as a strong selective pressure that eliminates B‐lineage‐committed subclones while sparing or selecting cells with myeloid potential.^7^, ^12^, ^17^, ^83^ The inflammatory microenvironment generated by immunotherapy may function as a biological amplifier, lowering the activation threshold for myeloid transcriptional programs in susceptible cells.^7^ Lineage conversion is then executed through downregulation of B‐cell TF networks (PAX5, EBF1), epigenetic and chromatin remodeling (CHD4/NuRD, enhancer reprogramming) and de‐repression and activation of myeloid TFs (PU.1, C/EBPα, C/EBPβ, and RUNX1).^15^, ^27^, ^36^, ^85^ A summary of these key points is illustrated in Figure 6.

**Figure 6:**
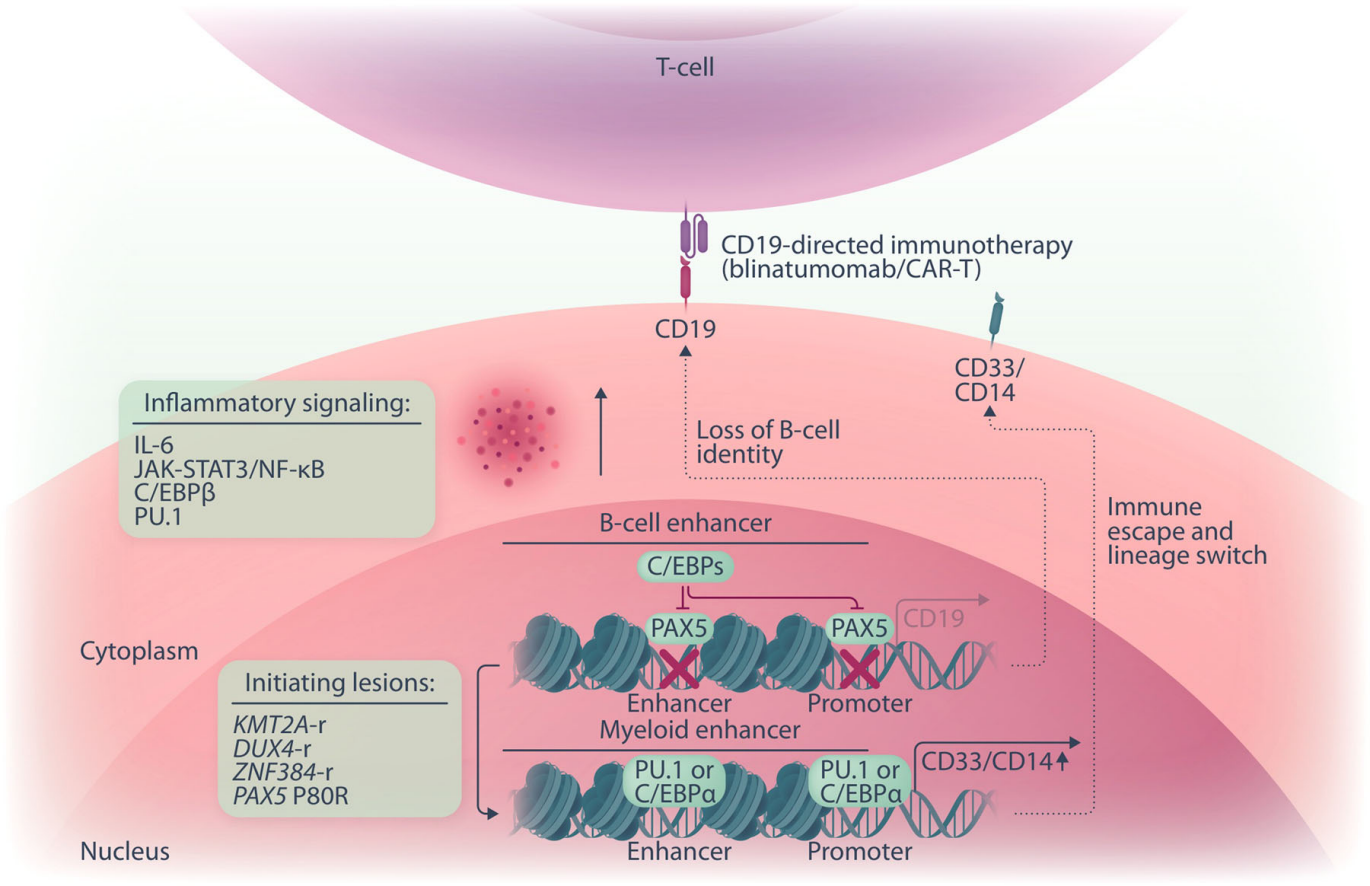
**Proposed mechanism of lineage switch (LS) in B‐lymphoblastic leukemia (B‐ALL).** Depicted in pink is a B‐ALL blast cell, and in purple, a T‐cell. Initiating lesions (*KMT2A*‐r, *DUX4*‐r, *ZNF384*‐r, and *PAX5* P80R) and the increased inflammatory signaling associated with CD19‐directed immunotherapy: interleukin‐6 (IL‐6), JAK–STAT3, and nuclear factor κ‐light‐chain‐enhancer of activated B‐cells (NF‐κB) signaling, with induction of C/EBPβ and PU.1. In the B‐cell state, PAX5 binds B‐lineage enhancer and promoter elements and maintains expression of B‐cell genes, including CD19. C/EBP factors repress PAX5 at these elements, which reduces B‐lineage transcription and silences CD19 (loss of B‐cell identity). At the same time, PU.1 and C/EBPα bind myeloid enhancer and promoter elements and activate myeloid genes, including CD33 and CD14. CD19‐directed immunotherapy (blinatumomab or CAR‐T cell) applies the selective pressure that favors cells which have lost CD19, permitting immune escape and LS to a CD33/CD14‐positive myeloid phenotype. CRS, cytokine release syndrome; r, rearrangement.

## CONCLUSION AND FUTURE PERSPECTIVES

Collectively, the clinical and biological evidence reviewed here suggests that lineage plasticity accounts for a spectrum of clinical manifestations ranging from transient LD and stable mixed‐phenotype states to complete LS with AML transformation.^7^, ^28^ Several important questions remain unresolved. It is still unclear how much LS results from selection of pre‐existing myeloid‐competent clones and how much from active reprogramming of B‐lymphoid cells. Both mechanisms may even occur simultaneously. Whole‐genome phylogenetic data from one ALL‐to‐AML cases support branching evolution from an early‐diverging clone.^36^ In contrast, shared IgH/TCR‐r between LS‐AML and the original B‐ALL indicates a lymphoid clonal origin. At present, we cannot reliably predict or prevent LS. We therefore suggest incorporating prospective LS monitoring into multicenter blinatumomab and CAR‐T cell trials to determine its true incidence and identify patients at risk. Such studies should combine extended standardized flow cytometry with longitudinal genomic clonal tracking and, in selected cases, single‐cell sequencing before and during therapy. Particular attention should be paid to leukemias with established lineage plasticity, including *KMT2A*‐r, *ZNF384*‐r, *DUX4*‐r, and *PAX5* P80R B‐ALL. Extended immunophenotyping, including CD371 as a potential surrogate marker of *DUX4*‐r B‐ALL,^109^ could help identify minor populations with cross‐lineage features.

The key clinical question is whether LS can be predicted before it occurs. Prospective monitoring could determine whether LS‐prone clones are already present before antigen‐directed therapy and whether their expansion under therapeutic pressure predicts subsequent LS. If validated, such populations could inform risk‐adapted surveillance and treatment decisions. Strategies targeting lineage plasticity, including epigenetic‐ or menin‐directed approaches, should be explored as potential means of preventing lineage conversion.

## AUTHOR CONTRIBUTIONS

**Katharina Daugs**: Investigation; writing—original draft; validation; visualization; writing—review and editing; data curation. **Anna Prazmo**: Investigation; writing—original draft; validation; visualization; writing—review and editing. **Anindita Roy**: Conceptualization; writing—original draft; writing—review and editing; supervision. **Arndt Borkhardt**: Conceptualization; writing—original draft; writing—review and editing; supervision.

## CONFLICT OF INTEREST STATEMENT

Arndt Borkhardt: Amgen Advisory Board.

## FUNDING

This research received funding by the following sources: K.D. was funded by Deutsche Forschungsgemeinschaft (DFG, German Research Foundation)‐Project‐ID: 564694041. A.P. was funded by an Oxford Cancer Centre DPhil in Cancer Sciences studentship. A.R. was funded by Wellcome Trust Clinical Researcher Career Development Fellowship (216632/Z/19/Z) and Medical Research Council (MRC,UK) Molecular Haematology Unit grant MC_UU_00029/7.

## Supporting information

Supporting Information.

## Data Availability

The data that support the findings of this study are available in the supplementary material of this article.
